# A comparative analysis of spider prey spectra analyzed through the next‐generation sequencing of individual and mixed DNA samples

**DOI:** 10.1002/ece3.8252

**Published:** 2021-10-19

**Authors:** Tingbang Yang, Xuhao Song, Xiaoqin Xu, Caiquan Zhou, Aimin Shi

**Affiliations:** ^1^ Key Laboratory of Southwest China Wildlife Resources Conservation (Ministry of Education) China West Normal University Nanchong China; ^2^ Institute of Ecology China West Normal University Nanchong China

**Keywords:** biological control, molecular gut content analysis, predation, prey spectrum, spider

## Abstract

As one of the most abundant predators of insects in terrestrial ecosystems, spiders have long received much attention from agricultural scientists and ecologists. Do spiders have a certain controlling effect on the main insect pests of concern in farmland ecosystems? Answering this question requires us to fully understand the prey spectrum of spiders. Next‐generation sequencing (NGS) has been successfully employed to analyze spider prey spectra. However, the high sequencing costs make it difficult to analyze the prey spectrum of various spider species with large samples in a given farmland ecosystem. We performed a comparative analysis of the prey spectra of *Ovia alboannulata* (Araneae, Lycosidae) using NGS with individual and mixed DNA samples to demonstrate which treatment was better for determining the spider prey spectra in the field. We collected spider individuals from tea plantations, and two treatments were then carried out: (1) The DNA was extracted from the spiders individually and then sequenced separately (DESISS) and (2) the DNA was extracted from the spiders individually and then mixed and sequenced (DESIMS). The results showed that the number of prey families obtained by the DESISS treatment was approximately twice that obtained by the DESIMS treatment. Therefore, the DESIMS treatment greatly underestimated the prey composition of the spiders, although its sequencing costs were obviously lower. However, the relative abundance of prey sequences detected in the two treatments was slightly different only at the family level. Therefore, we concluded that if our purpose were to obtain the most accurate prey spectrum of the spiders, the DESISS treatment would be the best choice. However, if our purpose were to obtain only the relative abundance of prey sequences of the spiders, the DESIMS treatment would also be an option. The present study provides an important reference for choosing applicable methods to analyze the prey spectra and food web compositions of animal in ecosystems.

## Graphical abstract

Individual DNA sample sequencing obtains the accurate prey spectrum of spiders. Mixed DNA sample sequencing underestimates the prey spectrum of spiders. Individual and mixed DNA sample sequencing obtains almost the same relative abundance of prey sequences of spiders.

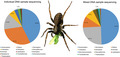

## 1 INTRODUCTION

Biological control involves the use of natural enemies to control pest species, and it has gained recognition as an essential component of integrated pest management (IPM) (Hoy & Herzog, 2012). Predatory natural enemies play key functional roles in biological control (Östman et al., 2003). Spiders are among the most abundant predators of insects in terrestrial ecosystems (Nyffeler & Benz, 1987). Nyffeler and Birkhofer (2017) estimated that the annual prey kill of the global spider community is in the range of 400–800 million metric tons, with insects and collembolans composing more than 90% of the captured prey. Approximately 49,361 spider species are currently known throughout the world (World Spider Catalog, 2021). Among them, many spider species have been reported in farmland ecosystems, such as tea plantations, cotton fields, and rice fields, and they represent the most abundant predatory arthropods (Song et al., 2020; Wang et al., 1996; Yang, Shi, et al., 2020; Zhao, 1995). This means that spiders can be used as biological control agents for agricultural pests. During the process, the prey spectrum of spiders in the farmland ecosystem of concern must be understood, and then, the spiders must be protected and effectively utilized for pest control. Therefore, it is important to develop a technique to accurately detect the prey spectrum of spiders in farmland ecosystems.

Spiders are fluid feeders, which makes it impossible to identify the species of prey that remain in the spider's gut with a microscopic examination approach (Piñol et al., 2014). Additionally, spiders are mostly very small in body size, and many species hunt nocturnally, making direct observations of predation events extremely challenging (Yang, Liu, Yuan, Zhang, Peng, et al., 2017). Molecular gut content analysis overcomes these challenges and has been successfully used to study spider predation events (Kennedy et al., 2019; King et al., 2008). The predation relationship between spiders and prey is determined by detecting specific DNA fragments of prey remains in the spider's gut. Conventional polymerase chain reaction (cPCR), quantitative PCR (qPCR), and next‐generation sequencing (NGS) technology have been used for molecular gut content analysis (Macías‐Hernández et al., 2018; Yang, Liu, et al., 2020). Among them, cPCR and qPCR are suitable for studying predation on a single or a few target prey based on prey‐specific primers (Yang, Liu, et al., 2020; Yang, Liu, Yuan, Zhang, Li, et al., 2017; Yang, Liu, Yuan, Zhang, Peng, et al., 2017). NGS is suitable for analyzing the prey composition of generalist predators (e.g., spiders) based on general primers for potential prey (Zhong et al., 2019). The DNA barcodes of various prey species remaining in the predator gut are sequenced by NGS technology, and the taxonomic classification of the prey can be determined by matching the sequence results to the DNA barcodes from a public database or to a prey DNA library specifically designed for the study.

To date, NGS technology has been successfully used to analyze the prey spectra of spiders (Piñol et al., 2014; Ramírez et al., 2021; Zhong et al., 2019). Although the cost of NGS is gradually decreasing, it is still relatively high (currently, it costs approximately 80 dollars per DNA sample to sequence the prey barcode region when use Illumina platform). This makes it difficult to analyze the prey spectrum of various spider species with large samples in a given farmland ecosystem using this technology. In previous studies, the DNA of field‐collected spiders has often been extracted individually and then sequenced separately for prey spectrum analysis (Cuff et al., 2021; Lafage et al., 2020; Toju & Baba, 2018). In this way, a large number of sequences can be obtained for the analysis of the prey spectrum, but the cost of sequencing is relatively high because the genomic DNA samples of multiple individuals of the same spider species are sequenced. The DNA of field‐collected spiders can also be extracted individually and then mixed and sequenced for prey spectrum analysis (Piñol et al., 2014). A variety of prey sequences per mixed DNA sample can be obtained using this treatment, and the sequencing costs of this approach are relatively low, with only a few mixed DNA samples. Therefore, our research question focuses mainly on whether an analysis of the spider's prey spectrum using NGS with mixed DNA samples can accurately characterize the prey spectrum. If so, this approach would considerably reduce the associated sequencing costs of this analysis.

*Ovia alboannulata* (Yin, Peng, Xie, Bao & Wang, 1997) (Araneae, Lycosidae) is a dominant spider species in tea plantations in Pujiang County, Chengdu city, Sichuan Province, China. This spider occurs throughout the year in tea plantations, especially from October to December (unpublished data). Therefore, it is readily available as a material for exploring the prey spectrum of spiders using NGS with individual and mixed DNA samples. Previous studies have shown that cytochrome oxidase I (COI), a barcode gene marker, can be effectively used to analyze the prey spectrum of insectivorous predators (Ammann et al., 2020; Cuff et al., 2021; Dušátková et al., 2020; King et al., 2015; Krehenwinkel et al., 2017). Therefore, we chose the COI gene for the identification of prey remains in the spider's gut. We collected *O*. *alboannulata* individuals from tea plantations. Two treatments were then used to analyze the spider's prey spectrum: (1) The DNA was extracted from the spiders individually and then sequenced separately (DESISS), and (2) the DNA was extracted from the spiders individually and then mixed and sequenced (DESIMS). Finally, the prey spectrum of *O*. *alboannulata* was obtained, and it was determined which treatment was better for determining the spider's prey spectrum.

## 2 MATERIALS AND METHODS

### 2.1 Sampling

The study site was located at a tea plantation in Pujiang County (103.37E; 30.19N), Chengdu city, Sichuan Province, China. *O*. *alboannulata* is a dominant spider species in this area. *O*. *alboannulata*, and its potential prey species were collected at the study site from October to December 2019. The specimens were collected by beating the canopy of *Camellia sinensis* with a 0.5‐m wooden stick (2 cm in diameter) above an insect net (50 cm in diameter). To avoid contamination, *O*. *alboannulata* and its potential prey species were placed in plastic bottles (200 ml) separately with 100% ethanol and stored at –20°C. All specimens were identified from the reference keys and catalogues provided by Zhang and Tan (2004), Song et al. (2020), and the World Spider Catalog (2021). After identification, individual number of *O*. *alboannulata* and its potential prey species were counted.

### 2.2 DNA extraction

**FIGURE 1:**
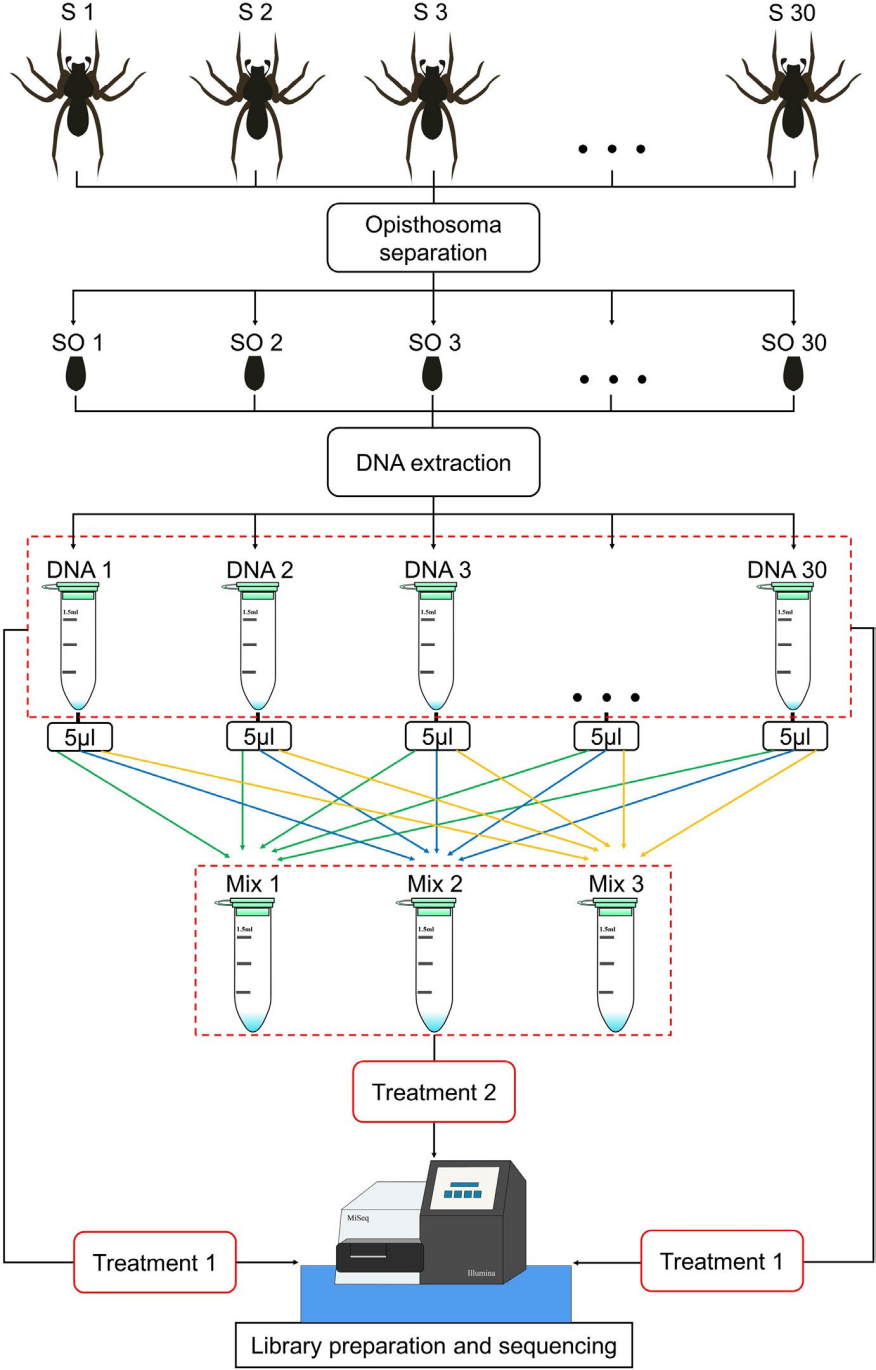
DNA extraction and processing for *Ovia alboannulata*. S, spider; SO, spider opisthosoma; Mix, a single pooled sample consisting of 5 μl from each of the 30 DNA samples

A total of 30 individuals of *O*. *alboannulata* (including juvenile and adult females, approximately 5 mm in body length) were randomly chosen and used for DNA extraction. Due to the spider gut's extensive bifurcations (Foelix, 2011), the gut is not easily dissected from the surrounding tissues. In the present study, only spider opisthosoma was used for DNA extraction because it has a higher concentration of prey DNA than the cephalothorax (Macías‐Hernández et al., 2018), and it can be used effectively for analyses of the spider's prey spectrum (Krehenwinkel et al., 2017). The opisthosomas were removed from the spiders using sterilized forceps and blades (Figure 1). To avoid contamination, each spider opisthosoma was cleaned with ultrapure water before extraction. The spider opisthosomas were then placed individually into 1.5‐ml microcentrifuge tubes. The genomic DNA in the opisthosomas was extracted individually using the 2 × CTAB method as described by Vallet et al. (2008). Ultrapure water was used a negative control for each extraction process. The DNA of each extraction was eluted in 50 μl of DNase‐free water. The quantity (Table S1) and quality (Figure S1) of the extracted DNA were measured using a NanoDrop ND‐1000 spectrophotometer (Thermo Fisher Scientific) and agarose gel electrophoresis, respectively. The DNA samples were stored at –20°C and later used for library preparation and sequencing.

### 2.3 DNA sample processing

The spider prey spectra were compared between the DESISS and DESIMS treatments. For the DESISS treatment, 30 extracted DNA samples were used directly for library preparation and sequencing (Figure 1, Treatment 1). For the DESIMS treatment, we prepared a single pooled sample consisting of 5 μl from each of the 30 DNA samples as described by Piñol et al. (2014). A total of 3 mixed DNA samples were used for library preparation and sequencing (Figure 1, Treatment 2).

### 2.4 Library preparation and sequencing

The prey DNA from the extracted DNA was amplified using the forward primer mlCOIintF (GGWACWGGWTGAACWGTWTAYCCYCC) and the reverse primer Fol‐degen‐rev (TANACYTCNGGRTGNCCRAARAAYCA) (Leray et al., 2013; Yu et al., 2012). The primers amplified a 363‐bp amplicon located within the COI barcode region and have been shown to amplify prey DNA from the spider gut efficiently (Krehenwinkel et al., 2017). Sample‐specific 7 bp barcodes (Table S2) were incorporated into the primers for multiplex sequencing. The individual and mixed DNA samples were amplified by a 2720 Thermal cycler instrument (Applied Biosystems) using the primers described above. Amplification was carried out in a final volume of 25 μl. Each tube contained 5 μl of Q5^®^ reaction buffer (5×), 5 μl of Q5^®^ High‐Fidelity GC buffer (5×), 0.25 μl of Q5^®^ High‐Fidelity DNA Polymerase (5 U/μl, New England Biolabs, USA), 2 μl (2.5 mM) of dNTPs, 1 μl (10 µM) of each forward and reverse primer, 2 μl of DNA template, and 8.75 μl of ddH_2_O. The thermal cycle consisted of an initial step of 5 min at 98°C, followed by 27 cycles of denaturation at 98°C for 30 s, annealing at 50°C for 30 s, extension at 72°C for 45 s, and a final extension step of 5 min at 72°C. Each run contained a negative control (without DNA template). PCR products were purified with VAHTSTM DNA Clean Beads (Vazyme) and quantified using the Quant‐iT PicoGreen dsDNA Assay Kit (Invitrogen, USA). Purification and quantification processes were performed according to the manufacturer's instructions. After the individual quantification (Table S3) step, PCR products were pooled in equimolar amounts, and paired‐end 2 × 250 bp sequencing was performed on the Illumina MiSeq platform (Illumina) with MiSeq Reagent Kit v3 (Illumina) at Shanghai Personal Biotechnology Co., Ltd.

### 2.5 Sequence analysis

All sequences were analyzed using QIIME2 (Version 2019.4) (Bolyen et al., 2019) according to the official tutorials, with slight modifications (https://docs.qiime2.org/2019.4/tutorials/). Briefly, raw sequencing reads that exactly matched the sample‐specific barcodes were assigned to respective samples and identified as valid sequences. The sequences were then merged, quality filtered, and dereplicated using the functions fastq_mergepairs, fastq_filter, and derep_fullength in VSEARCH software (Rognes et al., 2016). After chimera detection, the remaining high‐quality sequences were clustered into operational taxonomic units (OTUs) at 97% sequence identity by UCLUST (Edgar, 2010). A representative sequence was selected from each OTU using default parameters. Taxonomy was assigned to the OTUs using the classify‐sklearn naïve Bayes taxonomy classifier in the feature‐classifier plug‐in (Bokulich et al., 2018) against the NCBI database. An OTU table was further generated to record the relative abundance of each OTU in each sample and the taxonomy of the OTUs.

## 3 RESULTS

### 3.1 Sequence processing

A total of 4,136,851 raw sequences were obtained from the DESISS treatment, with 30 DNA samples. A total of 3,679,929 high‐quality sequences were obtained after the sequences were merged and filtered and chimeras were removed (Table S4). A total of 459,282 raw sequences were obtained from the DESIMS treatment, with 3 mixed DNA samples. A total of 417,380 high‐quality sequences were obtained after sequences were merged and filtered, and chimeras were removed (Table S5).

### 3.2 Taxonomy of the sequences

**FIGURE 2:**
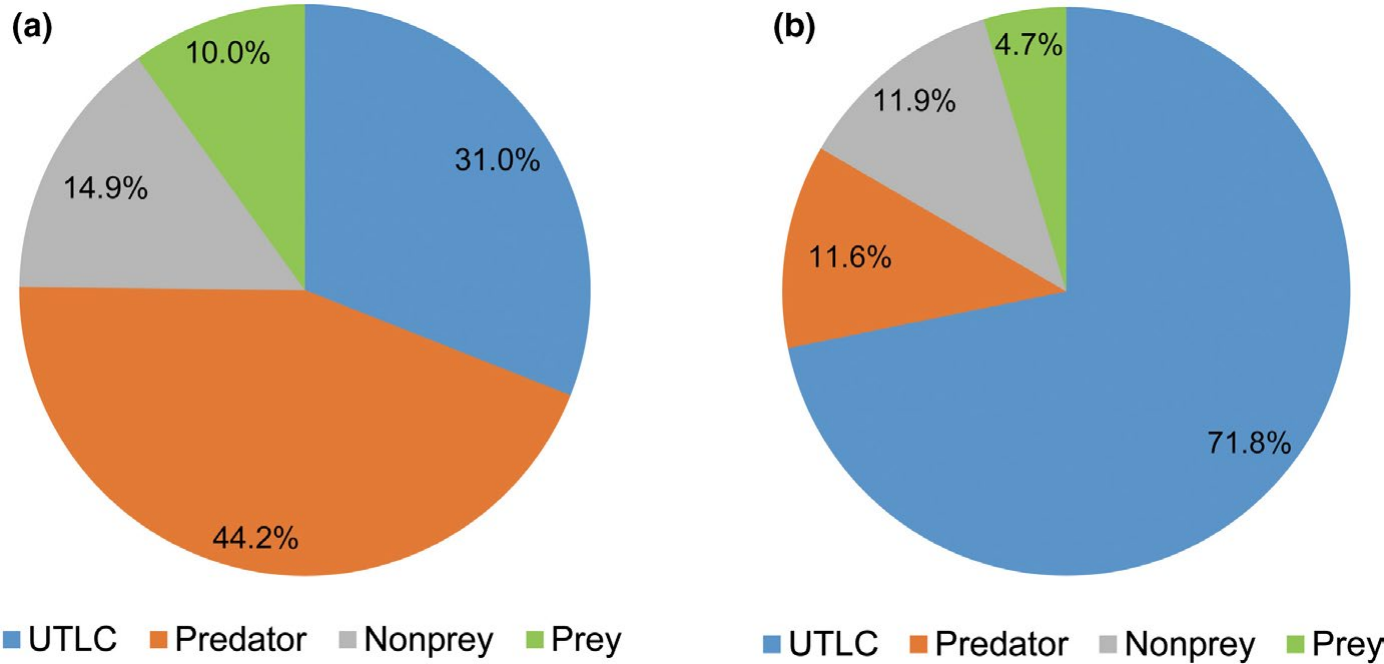
Sequence statistics after the sequences were identified using the classify‐sklearn naïve Bayes taxonomy classifier. (a) DESISS treatment; (b) DESIMS treatment; UTLC, unclassified to the taxonomic level of class. The relative abundance of sequence was shown in the pie chart by a percentage

For each DNA sample sequence, the high‐quality sequences were clustered into OTUs at 97% sequence identity. The representative sequence from each OTU was identified using the classify‐sklearn naïve Bayes taxonomy classifier. The results showed that the numbers of unclassified to the taxonomic level of class, predator, nonprey (including fungi, Chordata, aquatic taxa (Chrysophyta, Phaeophyta, Rhodophyta, Cnidaria, Echinodermata, Platyhelminthes, Rotifera, and some aquatic arthropods), soil‐dwelling taxa (Annelida), and some vertebrate parasite taxa (Rhabditida and Strongylida)) and prey sequences were 1,140,705, 1,625,713, 547,311, and 366,200, respectively, from the 30 DNA samples sequenced in the DESISS treatment, accounting for 31.0%, 44.2%, 14.9%, and 10.0% of the total sequences, respectively (Figure 2a). The numbers of unclassified to the taxonomic level of class, predator, nonprey (including fungi, Chordata, aquatic taxa (Phaeophyta and some aquatic arthropods), soil‐dwelling taxa (Annelida), and some vertebrate parasite taxa (Rhabditida and Strongylida)) and prey sequences were 299,684, 48,355, 49,691, and 19,650, respectively, with the 3 mixed DNA samples sequenced from the DESIMS treatment, accounting for 71.8%, 11.6%, 11.9%, and 4.7% of the total sequences, respectively (Figure 2b).

### 3.3 Comparison of the *O. alboannulata* prey compositions obtained from the DESISS and DESIMS treatments

**FIGURE 3:**
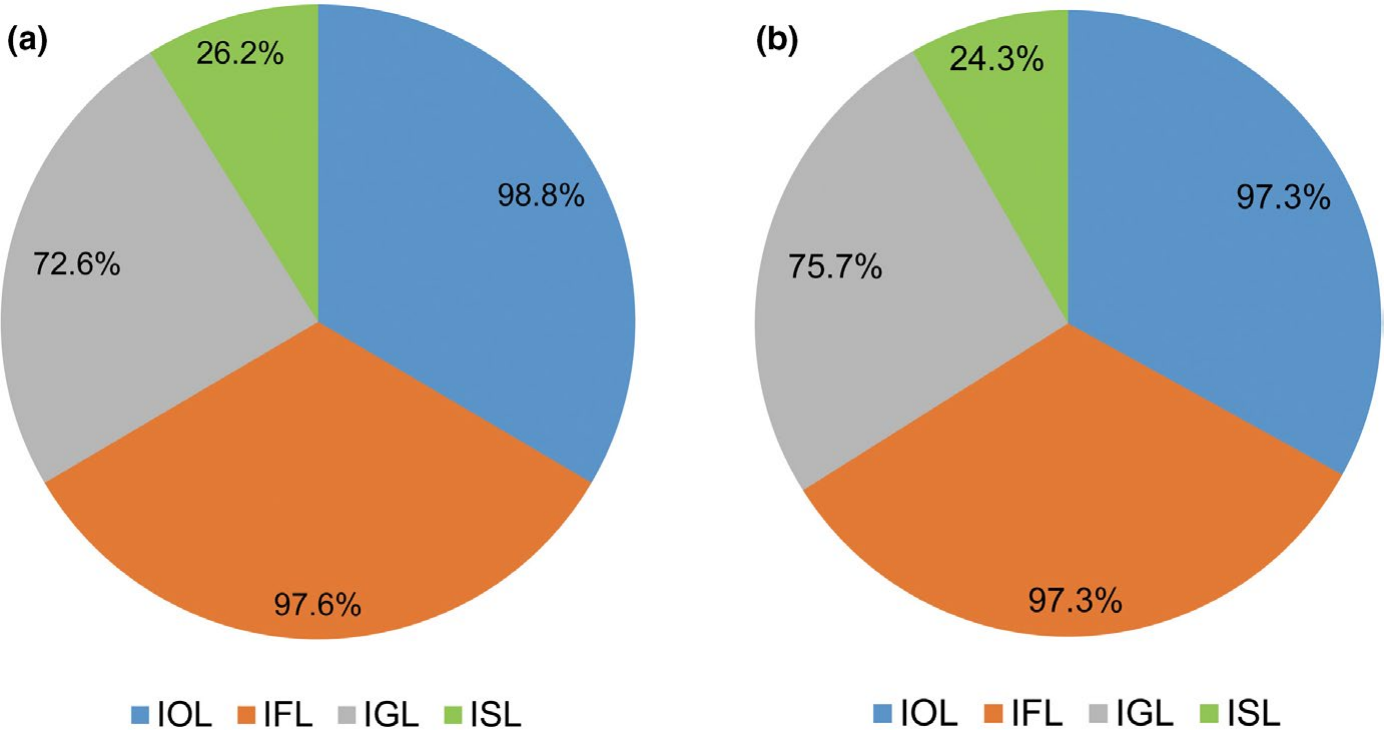
OTU statistics after the prey sequences were identified using the classify‐sklearn naïve Bayes taxonomy classifier. (a) DESISS treatment; (b) DESIMS treatment; IOL, Identified to the order levels; IFL, Identified to the family levels; IGL, Identified to the genus levels; ISL, Identified to the species levels. The relative abundance of OTU was shown in the pie chart by a percentage

A total of 366,200 sequences were used in the spider prey spectrum annotation after the DESISS treatment. A total of 84 OTUs were obtained. Among them, 83, 82, 61, and 22 OTUs were identified to the order, family, genus, and species levels, respectively, accounting for 98.8%, 97.6%, 72.6%, and 26.2% of the total OTU number, respectively (Figure 3a). A total of 3 classes, 13 orders, 55 families, 57 genera, and 22 species of prey were identified (Table S6). A total of 19,650 sequences were used in the spider prey spectrum annotation after the DESIMS treatment. A total of 37 OTUs were obtained. Among them, 36, 36, 28, and 9 OTUs were identified to the order, family, genus, and species levels, respectively, accounting for 97.3%, 97.3%, 75.7%, and 24.3% of the total OTU number, respectively (Figure 3b). A total of 3 classes, 11 orders, 29 families, 28 genera, and 9 species of prey were identified (Table S6).

**FIGURE 4:**
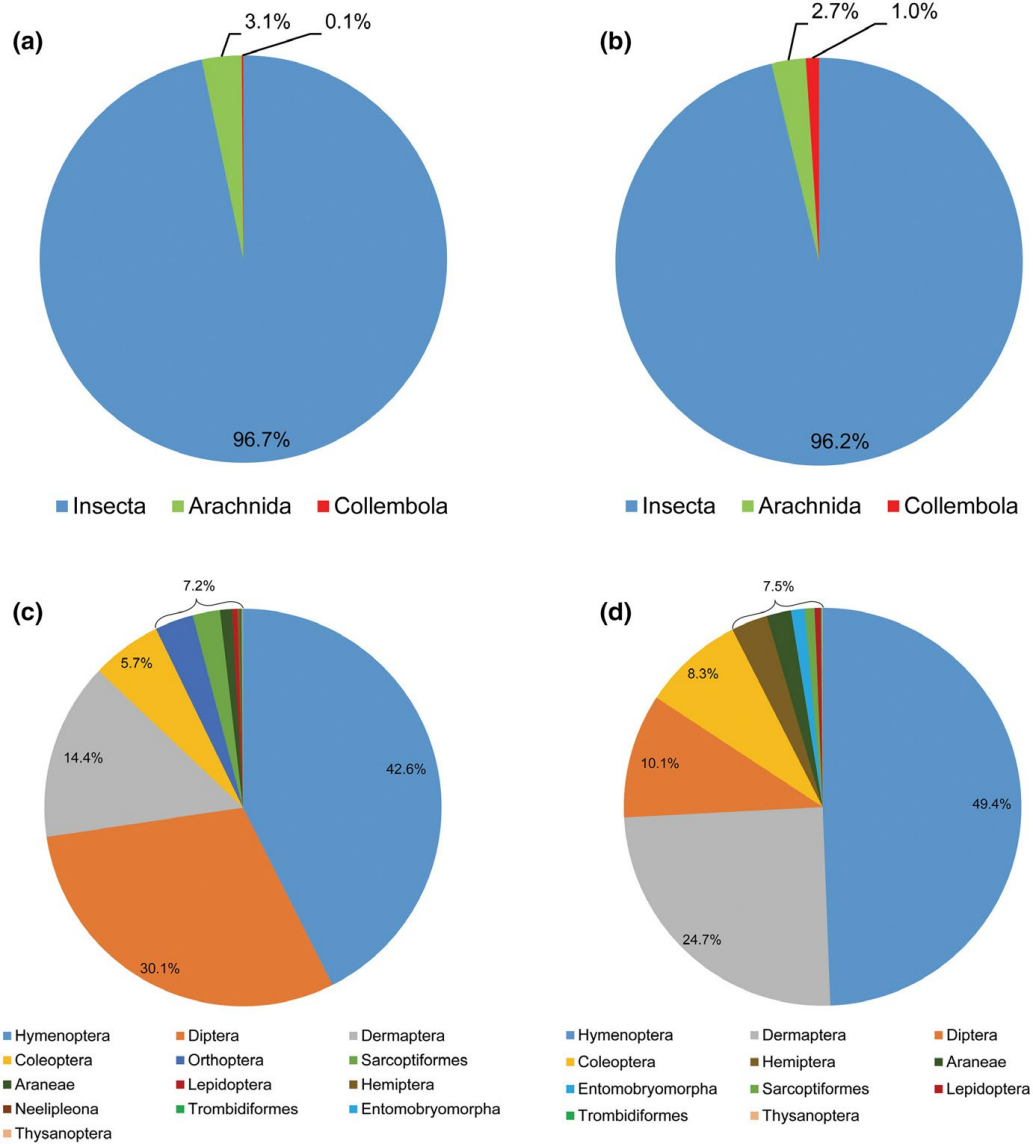
The prey spectra of *Ovia alboannulata* obtained by the DESISS and DESIMS treatments at the class and order level. (a, c) DESISS treatment; (b, d) DESIMS treatment. The relative abundance of prey sequences was shown in the pie chart by a percentage

The prey composition and relative abundance of prey sequences were compared between the two treatments at the class, order, and family levels. The results showed that the prey composition and relative abundance of prey sequences obtained with the DESISS treatment were similar to those obtained with the DESIMS treatment at the class level. The prey composition included Insecta, Arachnida, and Collembola, of which Insecta was the most abundant, accounting for more than 90% of the total prey sequences (Figure 4a,b). There were slight differences in prey composition between the two treatments at the order level. The number of orders obtained from the DESISS treatment was 2 more than that obtained from the DESIMS treatment (Figure 4c,d). However, the relative abundance of prey sequences obtained from the DESISS treatment was similar to that obtained from the DESIMS treatment at the order level. The prey spectra obtained by the two treatments mainly included Hymenoptera, Diptera, Dermaptera, and Coleoptera, which accounted for more than 90% of the total prey sequences (Figure 4c,d). The prey compositions were obviously different between the two treatments at the family level. The number of families obtained from the DESISS treatment was 26 more than that obtained from the DESIMS treatment (Table 1). However, there were slight differences in relative abundance of prey sequences between the two treatments at the family level. The prey spectrum obtained from the DESISS treatment mainly included Formicidae, Forficulidae, and Sciaridae (insects), accounting for 67.3% of the total prey sequences. The prey spectrum obtained from the DESIMS treatment mainly included Formicidae and Forficulidae (insects), accounting for 72.4% of the total prey sequences (Table 1).

**TABLE 1:** The prey spectrum of *Ovia alboannulata* obtained by the DESISS and DESIMS treatments at the family level

Family	DESISS		DESIMS	
	Number of sequences	%	Number of sequences	%
Formicidae	137,174	39.5	8873	47.7
Forficulidae	50,594	14.6	4600	24.7
Sciaridae	45,868	13.2	524	2.8
Drosophilidae	15,111	4.4	918	4.9
Asilidae	15,097	4.3	1	0.01
Scarabaeidae	14,486	4.2	0	0
Phoridae	12,607	3.6	123	0.7
Braconidae	11,448	3.3	278	1.5
Acrididae	11,027	3.2	0	0
Elmidae	4613	1.3	1348	7.3
Dolichopodidae	4139	1.2	0	0
Oribatellidae	2916	0.8	28	0.2
Cecidomyiidae	2892	0.8	190	1.0
Oppiidae	2815	0.8	122	0.7
Anthomyiidae	2475	0.7	11	0.1
Muscidae	2362	0.7	37	0.2
Theridiidae	2075	0.6	157	0.8
Geometridae	1228	0.4	92	0.5
Scutoverticidae	1203	0.3	0	0
Chironomidae	934	0.3	0	0
Thomisidae	909	0.3	139	0.7
Phenopelopidae	856	0.2	0	0
Chrysomelidae	564	0.2	0	0
Eulophidae	524	0.2	27	0.1
Aleyrodidae	349	0.1	357	1.9
Cicadellidae	307	0.1	66	0.4
Neelidae	302	0.1	0	0
Culicidae	297	0.1	34	0.2
Lycosidae	289	0.1	15	0.1
Tabanidae	143	0.04	0	0
Autostichidae	142	0.04	0	0
Eriophyidae	141	0.04	0	0
Thripidae	131	0.04	11	0.1
Scatopsidae	130	0.04	0	0
Eupodidae	125	0.04	0	0
Latridiidae	116	0.03	0	0
Cerambycidae	115	0.03	0	0
Entomobryidae	110	0.03	205	1.1
Dermestidae	97	0.03	0	0
Salticidae	92	0.03	57	0.3
Gracillariidae	78	0.02	0	0
Tomoceridae	73	0.02	0	0
Aphelinidae	71	0.02	0	0
Tachinidae	59	0.02	31	0.2
Coccinellidae	58	0.02	0	0
Tetragnathidae	53	0.02	0	0
Mycetophilidae	48	0.01	0	0
Aphididae	38	0.01	0	0
Sphaeroceridae	26	0.01	0	0
Bdellidae	18	0.005	0	0
Pisauridae	18	0.005	0	0
Psychodidae	14	0.004	12	0.1
Araneidae	13	0.004	0	0
Oedemeridae	2	0.001	186	1.0
Scydmaenidae	2	0.001	0	0
Reduviidae	0	0	127	0.7
Tarsonemidae	0	0	17	0.1

Note

The relative abundance of prey sequences was shown in the table by a percentage, the same below.

## 4 DISCUSSION

As among the most abundant predators of insects in terrestrial ecosystems, spiders have long received much attention from agricultural scientists and ecologists (Marc & Canard, 1997; Nyffeler & Benz, 1987; Nyffeler & Sunderland, 2003). Do spiders have a certain controlling effect on the main insect pests of concern in farmland ecosystems? Answering this question requires us to develop an applicable method for evaluating the control effect of spiders on target insect pests. The dominance of spiders, the population dynamics between spiders and insect pests, and the predation rates and predation numbers of spiders on target insect pests are often used as evaluation indicators of which spiders control target insect pests (Yang, Liu, Yuan, Zhang, Peng, et al., 2017). Among them, the former two indices often lack direct evidence for determining whether spiders prey on target insect pests in the field. Therefore, it is particularly important to determine the predation relationship between spiders and target insect pests and then qualitatively and quantitatively evaluate the predation of spiders on the target insect pests. The specific characteristics of spiders, such as their mostly very small body size, diverse predation strategies, euryphagy, fluid feeding habits, and nocturnal hunting (in many species) (Foelix, 2011), make it difficult to study spider predation events, although NGS has been successfully employed to analyze spider prey spectra (Piñol et al., 2014). However, considering the high cost of this technology, we performed a comparative analysis of spider prey spectra using NGS with individual and mixed DNA samples to demonstrate which treatment was better for determining the spider's prey spectrum in the field.

We chose COI as the barcode gene marker for the prey remains in the spider's gut based on the large number of COI genes of insects in GenBank (Barr et al., 2012; Hogg & Hebert, 2004; Jung et al., 2011; Monaghan et al., 2005). The field‐collected spiders were extracted genomic DNA and sequenced using a universal primer (Krehenwinkel et al., 2017). A total of 3,679,929 and 417,380 high‐quality sequences were obtained by individual and mixed DNA sample sequencing, respectively (Tables S4 and S5). The taxonomy of the prey species showed that large numbers of sequences were not classified to class level, accounting for 31.0% and 71.8% of the total sequences, respectively. The identification of these sequences relies on the existence of the COI genes of prey species in the GenBank database (Zhong et al., 2019). Therefore, more work needs to be carried out on the barcoding of organisms found in tea plantations to improve our ability to fully identify these sequences (Piñol et al., 2014).

Both the DESISS and DESIMS treatments revealed a certain number of nonprey sequences, accounting for 14.9% and 11.9% of the total sequences, respectively. These sequences were likely to be introduced during the library preparation and sequencing processes, as PCR can amplify minute quantities of contaminant DNA due to its high sensitivity (King et al., 2008). The primer pairs (mlCOIintF/Fol‐degen‐rev) are universal primers for the COI gene from mitochondria. Therefore, it can amplify many organisms with mitochondrial genes. Various organisms have been sequenced by sequencing companies, and the DNA sequences of these organisms can contaminate the instruments, reagents, handlers, and environment during the sequencing process (Adams et al., 2015; Salter et al., 2014; Weiss et al., 2014). Some studies have shown that contamination is a common phenomenon when using NGS to analyze predator–prey spectra (Ando et al., 2018). Therefore, the sequencing results need to be interpreted appropriately considering the inevitable contamination, and then, the introduced contamination sequences should be removed. In the present study, in order to remove nonprey sequences, we considered the dietary characteristics of the spider; that is, it usually feeds on insects and collembolans (Nyffeler & Birkhofer, 2017). These nonprey sequences belonged to fungi, Chordata, aquatic taxa (Chrysophyta, Phaeophyta, Rhodophyta, Cnidaria, Echinodermata, Platyhelminthes, Rotifera, and some aquatic arthropods), soil‐dwelling taxa (Annelida), and some vertebrate parasite taxa (Rhabditida and Strongylida).

Genomic DNA from the whole opisthosoma of the spider was extracted for sequencing analysis. Similar to the results of Piñol et al. (2014), the DESISS and DESIMS treatments both obtained many sequences of predators themselves, accounting for 44.2% and 11.6% of the total sequences, respectively. Universal primer pairs (mlCOIintF/Fol‐degen‐rev) can amplify the COI gene of prey remaining in the spider gut as well as in the spider itself. Toju and Baba (2018) designed spider‐specific blocking primers to reduce the sequence amplification of the spider itself. However, the results showed that the majority of sequencing reads obtained represented spiders rather than their prey. Therefore, blocking primers cannot completely prevent the amplification of the DNA of the predators themselves. In addition, blocking primers may lead to the risk of nonspecific coblocking when prey and predators are phylogenetically close (Piñol et al., 2014). Our results showed that *O*. *alboannulata* preys on some spider species in Lycosidae, Araneidae, Pisauridae, Salticidae, Tetragnathidae, and Theridiidae (Table S6), that is, intraguild predation (Petráková et al., 2016). Therefore, these prey species could have been blocked if blocking primers had been used. In the present study, we directly removed the sequences of predators themselves from the species taxonomy results without using blocking primers. The results showed that only 10.0% and 4.7% of the total sequences obtained from the DESISS and DESIMS treatments, respectively, were from spider prey species. However, these sequences still provided abundant information about the prey of this spider.

A total of 366,200 and 19,650 prey sequences were used in the spider prey spectrum annotation after the DESISS treatment and DESIMS treatment, respectively. The results showed that, although most of the OTUs were not identified to the species level. However, the total OTU number obtained by the DESISS treatment was obviously greater than that obtained by the DESIMS treatment. Therefore, compared with the DESIMS treatment, the DESISS treatment obtained a broader range of the prey spectrum of *O*. *alboannulata*.

The prey composition and relative abundance of prey sequences were compared between the two treatments at the class, order, and family levels. As shown in Table 1, prey with fewer sequences could be detected using the DESISS treatment, while some prey with fewer sequences could not be detected using the DESIMS treatment. The DESIMS treatment increased the prey abundance in a single DNA sample. However, some prey DNA that was present in small quantities was likely to be severely diluted after mixing the DNA samples, leading to false‐negative results from sequencing.

Zhong et al. (2019) analyzed the prey composition of *Pardosa pseudoannulata* (Araneae, Lycosidae) using NGS. The results showed that Coleoptera and Diptera were the most abundant prey of *P*. *pseudoannulata*. Lafage et al. (2020) analyzed the prey composition of 4 genera (*Hygrolycosa*, *Pardosa*, *Piratula*, *Trochosa*) of Lycosidae using NGS. The results showed that Diptera, Hemiptera, and Hymenoptera were the most abundant prey of these lycosid species. In the present study, the prey spectrum of *O*. *alboannulata* was analyzed using NGS with individual and mixed DNA samples. The results showed that Hymenoptera, Diptera, Dermaptera, and Coleoptera were the most abundant prey of this spider, which is consistent with the feeding characteristics of lycosid spiders. In addition, our results also showed that the prey species detected by the two treatments was consistent with the diversity of potential prey of *O*. *alboannulata* in study regions (Table S7). These results indicated that the NGS technology was reliable for studying the prey composition of predators. Our results showed that, compared with DESIMS treatment, DESISS treatment could obtain more accurate prey composition of spiders. In addition, DESISS treatment could be used to calculate the positive rate and relative abundance of the target DNA fragments of prey (Table 2). These data have often been used to evaluate the control effect of predators on target insect pests (Yang, Liu, Yuan, Zhang, Li, et al., 2017; Yang, Liu, Yuan, Zhang, Peng, et al., 2017). However, both treatments obtained almost the same relative abundance of prey sequences of spiders. Therefore, we suggest to choose the applicable method for the analysis of spider prey spectra according to our experimental purpose.

**TABLE 2:** The positive rate and relative abundance of the target DNA fragments of 5 main tea pests calculated with the DESISS treatment

Species	Number of tested spiders	Number of positive spiders	Positive rates (%)	Number of sequences	%
*Empoasca onukii*	30	2	6.7	226	0.06
*Aleurocanthus* sp.	30	5	16.7	349	0.10
*Ectropis grisescens*	30	3	10.0	134	0.04
*Caloptilia theivora*	30	1	3.3	78	0.02
*Dendrothrips minowai*	30	4	13.3	97	0.03

## 5 CONCLUSIONS

The present study showed that the prey compositions obtained were obviously different between the DESISS and DESIMS treatments at the family level. The number of prey families obtained by the DESISS treatment was approximately twice that obtained by the DESIMS treatment. Therefore, the DESIMS treatment greatly underestimated the prey composition of the spiders, although it was obviously less expensive than the DESISS treatment. However, the relative abundance of prey sequences detected in the two treatments was slightly different only at the family level. Therefore, to obtain the most accurate spider prey spectrum, the DESISS treatment is the best choice. However, to obtain only the relative abundance of prey sequences of spiders, the DESIMS treatment is also an option. Our results also showed that the DESISS treatment could not only obtain the prey composition of the spiders but could also be used to calculate the positive rate and relative abundance of the target DNA fragments of prey (Table 2). These data are useful for evaluating the potential of spiders to control target insect pests. The present study provides an important reference for use in choosing applicable methods to analyze spider prey spectra in farmland ecosystems.

## CONFLICT OF INTEREST

The authors declare no competing or financial interests.

## AUTHOR CONTRIBUTIONS

**Tingbang Yang:** Conceptualization (lead); Formal analysis (equal); Funding acquisition (equal); Investigation (equal); Methodology (equal); Supervision (equal); Writing‐original draft (equal); Writing‐review & editing (lead). **Xuhao Song:** Formal analysis (equal); Funding acquisition (equal); Investigation (equal); Methodology (equal); Writing‐original draft (equal); Writing‐review & editing (equal). **Xiaoqin Xu:** Investigation (equal); Writing‐original draft (equal). **Caiquan Zhou:** Supervision (equal); Writing‐original draft (equal); Writing‐review & editing (equal). **Aimin Shi:** Supervision (equal); Writing‐original draft (equal); Writing‐review & editing (equal).

## Supporting information

Supplementary Material

## Data Availability

Raw sequences are available online on Dryad repository (https://doi.org/10.5061/dryad.x3ffbg7k4).
