# Supplementary material for: A comparative analysis of spider prey spectra analyzed through the next‐generation sequencing of individual and mixed DNA samples
Source: Ecol Evol. 2021 Oct 19;11(21):15444–54. doi: 10.1002/ece3.8252 (PMC8571630; doi:10.1002/ece3.8252)
Supplement: Supplementary file 1 — Supplementary Material [file ECE3-11-15444-s001.docx]

**Supplementary material:**

**“A comparative analysis of spider prey spectra analyzed through the next-generation sequencing of individual and mixed DNA samples”**

Yang et al.

**Supplementary Tables**

**Table S1.** The quantity of extracted DNA from *O. alboannulata*. The quantity was measured using a NanoDrop ND-1000 spectrophotometer.

| Sample ID | Sample type | Volume (μl) | Concentration (ng/μl) | OD260/280 | OD260/230 |
| --- | --- | --- | --- | --- | --- |
| OVA_01 | DNA | 50 | 318.60 | 1.89 | 1.87 |
| OVA_02 | DNA | 50 | 307.60 | 1.92 | 1.74 |
| OVA_03 | DNA | 50 | 406.20 | 1.86 | 1.60 |
| OVA_04 | DNA | 50 | 140.40 | 1.69 | 1.59 |
| OVA_05 | DNA | 50 | 176.50 | 1.90 | 1.87 |
| OVA_06 | DNA | 50 | 175.00 | 1.90 | 1.90 |
| OVA_07 | DNA | 50 | 229.90 | 1.89 | 1.87 |
| OVA_08 | DNA | 50 | 222.30 | 1.91 | 1.85 |
| OVA_09 | DNA | 50 | 209.40 | 1.91 | 1.90 |
| OVA_10 | DNA | 50 | 283.00 | 1.91 | 1.89 |
| OVA_11 | DNA | 50 | 304.60 | 1.89 | 1.98 |
| OVA_12 | DNA | 50 | 668.60 | 1.89 | 1.83 |
| OVA_13 | DNA | 50 | 309.60 | 1.90 | 1.83 |
| OVA_14 | DNA | 50 | 271.90 | 1.91 | 1.89 |
| OVA_15 | DNA | 50 | 249.10 | 1.91 | 1.87 |
| OVA_16 | DNA | 50 | 328.70 | 1.78 | 1.34 |
| OVA_17 | DNA | 50 | 326.30 | 1.92 | 1.52 |
| OVA_18 | DNA | 50 | 274.90 | 1.92 | 1.88 |
| OVA_19 | DNA | 50 | 192.30 | 1.90 | 1.84 |
| OVA_20 | DNA | 50 | 394.10 | 1.87 | 1.81 |
| OVA_21 | DNA | 50 | 250.00 | 1.89 | 1.98 |
| OVA_22 | DNA | 50 | 320.30 | 1.89 | 1.95 |
| OVA_23 | DNA | 50 | 215.70 | 1.92 | 1.89 |
| OVA_24 | DNA | 50 | 304.10 | 1.89 | 1.91 |
| OVA_25 | DNA | 50 | 291.20 | 1.89 | 1.77 |
| OVA_26 | DNA | 50 | 327.10 | 1.90 | 1.93 |
| OVA_27 | DNA | 50 | 207.20 | 1.89 | 1.87 |
| OVA_28 | DNA | 50 | 622.20 | 1.88 | 1.80 |
| OVA_29 | DNA | 50 | 279.60 | 1.91 | 2.05 |
| OVA_30 | DNA | 50 | 217.00 | 1.91 | 1.91 |
| MIX 1 | DNA | 50 | 293.40 | 1.88 | 1.78 |
| MIX 2 | DNA | 50 | 293.00 | 1.88 | 1.80 |
| MIX 3 | DNA | 50 | 297.10 | 1.87 | 1.81 |

**Table S2.** Sample-specific 7 bp barcodes.

| Sample ID | Sample type | Barcode |
| --- | --- | --- |
| OVA_01 | DNA | CTTGAGT |
| OVA_02 | DNA | CTCACGA |
| OVA_03 | DNA | CTCAGAC |
| OVA_04 | DNA | CTTGAGT |
| OVA_05 | DNA | CTCACGA |
| OVA_06 | DNA | CTCAGAC |
| OVA_07 | DNA | CTCTCAG |
| OVA_08 | DNA | CTCTGTA |
| OVA_09 | DNA | CTGATGT |
| OVA_10 | DNA | CTGTAGA |
| OVA_11 | DNA | GAACACT |
| OVA_12 | DNA | GATCTCA |
| OVA_13 | DNA | GACATCT |
| OVA_14 | DNA | GACAGTA |
| OVA_15 | DNA | GACTACA |
| OVA_16 | DNA | GACTCAC |
| OVA_17 | DNA | GAGTCGT |
| OVA_18 | DNA | GTACAGA |
| OVA_19 | DNA | GTAGTCT |
| OVA_20 | DNA | GTCACAT |
| OVA_21 | DNA | GTCTAGT |
| OVA_22 | DNA | GTCGTGA |
| OVA_23 | DNA | GTGAACA |
| OVA_24 | DNA | GTGACTC |
| OVA_25 | DNA | GTGTCAA |
| OVA_26 | DNA | AACTGTC |
| OVA_27 | DNA | AACGTGT |
| OVA_28 | DNA | CTTGAGT |
| OVA_29 | DNA | CTCACGA |
| OVA_30 | DNA | CTCAGAC |
| MIX 1 | DNA | CTGTAGA |
| MIX 2 | DNA | GAACACT |
| MIX 3 | DNA | GATCTCA |

**Table S3.** The quantity of PCR products. The quantity were measured using Quant-iT PicoGreen dsDNA Assay Kit (Invitrogen, USA).

| Sample ID | Amplification region | Fragment size (bp) | Concentration (ng/μl) |
| --- | --- | --- | --- |
| OVA_01 | COI | 363 | 1.92 |
| OVA_02 | COI | 363 | 1.82 |
| OVA_03 | COI | 363 | 2.28 |
| OVA_04 | COI | 363 | 1.50 |
| OVA_05 | COI | 363 | 0.75 |
| OVA_06 | COI | 363 | 1.50 |
| OVA_07 | COI | 363 | 1.83 |
| OVA_08 | COI | 363 | 1.73 |
| OVA_09 | COI | 363 | 2.52 |
| OVA_10 | COI | 363 | 2.44 |
| OVA_11 | COI | 363 | 2.58 |
| OVA_12 | COI | 363 | 2.04 |
| OVA_13 | COI | 363 | 1.62 |
| OVA_14 | COI | 363 | 1.54 |
| OVA_15 | COI | 363 | 3.88 |
| OVA_16 | COI | 363 | 0.88 |
| OVA_17 | COI | 363 | 0.55 |
| OVA_18 | COI | 363 | 1.17 |
| OVA_19 | COI | 363 | 0.64 |
| OVA_20 | COI | 363 | 2.24 |
| OVA_21 | COI | 363 | 2.96 |
| OVA_22 | COI | 363 | 1.94 |
| OVA_23 | COI | 363 | 3.63 |
| OVA_24 | COI | 363 | 2.03 |
| OVA_25 | COI | 363 | 2.32 |
| OVA_26 | COI | 363 | 1.95 |
| OVA_27 | COI | 363 | 4.27 |
| OVA_28 | COI | 363 | 3.15 |
| OVA_29 | COI | 363 | 1.57 |
| OVA_30 | COI | 363 | 0.88 |
| MIX 1 | COI | 363 | 1.64 |
| MIX 2 | COI | 363 | 2.06 |
| MIX 3 | COI | 363 | 1.17 |

**Table S4.** Sequence statistics after the sequences were merged and filtered and chimeras were removed. Thirty DNA samples were sequenced from the DESISS treatment.

|  | Sample ID | Number of sequence | | | |
| --- | --- | --- | --- | --- | --- |
|  |  | Raw | Merged | Filtered | Non-chimeric |
|  | OVA_01 | 122792 | 115210 | 114159 | 109860 |
|  | OVA_02 | 151513 | 137217 | 135618 | 130502 |
|  | OVA_03 | 120965 | 114012 | 112595 | 109913 |
|  | OVA_04 | 144310 | 120778 | 117209 | 117009 |
|  | OVA_05 | 144348 | 133618 | 128796 | 128044 |
|  | OVA_06 | 141374 | 130606 | 125948 | 125448 |
|  | OVA_07 | 151682 | 141319 | 136623 | 136279 |
|  | OVA_08 | 146993 | 136954 | 133247 | 132688 |
|  | OVA_09 | 198789 | 183410 | 178481 | 175930 |
|  | OVA_10 | 131519 | 122314 | 118747 | 118428 |
|  | OVA_11 | 129667 | 119952 | 116001 | 114292 |
|  | OVA_12 | 133131 | 122949 | 119103 | 118369 |
|  | OVA_13 | 129585 | 118085 | 114645 | 113914 |
|  | OVA_14 | 133344 | 124043 | 120245 | 119422 |
|  | OVA_15 | 128849 | 120331 | 116829 | 116490 |
|  | OVA_16 | 128178 | 118229 | 113982 | 113672 |
|  | OVA_17 | 127765 | 116681 | 112682 | 112453 |
|  | OVA_18 | 124627 | 115745 | 111738 | 111497 |
|  | OVA_19 | 151774 | 142633 | 138672 | 138522 |
|  | OVA_20 | 221095 | 199321 | 193503 | 192585 |
|  | OVA_21 | 132609 | 124137 | 120481 | 120064 |
|  | OVA_22 | 125260 | 114268 | 111175 | 110848 |
|  | OVA_23 | 128379 | 119028 | 115173 | 114987 |
|  | OVA_24 | 124163 | 115962 | 112696 | 112370 |
|  | OVA_25 | 125612 | 117547 | 114246 | 113395 |
|  | OVA_26 | 123984 | 116072 | 112635 | 112286 |
|  | OVA_27 | 119505 | 111963 | 108742 | 108319 |
|  | OVA_28 | 123666 | 115593 | 112339 | 112175 |
|  | OVA_29 | 128765 | 119101 | 114804 | 114458 |
|  | OVA_30 | 142608 | 130829 | 125835 | 125710 |
| Total |  | 4136851 | 3817907 | 3706949 | 3679929 |

**Table S5.** Sequence statistics after sequences were merged and filtered and chimeras were removed. Three mixed DNA samples were sequenced from the DESIMS treatment.

|  | Sample ID | Number of sequence | | | |
| --- | --- | --- | --- | --- | --- |
|  |  | Raw | Merged | Filtered | Non-chimeric |
|  | MIX 1 | 148666 | 139903 | 136534 | 135851 |
|  | MIX 2 | 154735 | 144807 | 140534 | 139405 |
|  | MIX 3 | 155881 | 146690 | 142893 | 142124 |
| Total |  | 459282 | 431400 | 419961 | 417380 |

**Table S6.** The prey spectra of *O. alboannulata* obtained from the DESISS and DESIMS treatments.

| Class | Order | Family | Genus | Species | Number of sequence | |
| --- | --- | --- | --- | --- | --- | --- |
|  |  |  |  |  | DESISS | DESIMS |
| Arachnida | Araneae | Araneidae | *Cyclosa* | *Cyclosa argenteoalba* | 13 | 0 |
| Arachnida | Araneae | Lycosidae |  |  | 278 | 15 |
| Arachnida | Araneae | Lycosidae | *Alopecosa* |  | 6 | 0 |
| Arachnida | Araneae | Lycosidae | *Trochosa* |  | 5 | 0 |
| Arachnida | Araneae | Pisauridae | *Dolomedes* |  | 18 | 0 |
| Arachnida | Araneae | Salticidae | *Evarcha* |  | 9 | 0 |
| Arachnida | Araneae | Salticidae | *Telamonia* |  | 83 | 57 |
| Arachnida | Araneae | Tetragnathidae | *Guizygiella* |  | 20 | 0 |
| Arachnida | Araneae | Tetragnathidae | *Tetragnatha* | *Tetragnatha maxillosa* | 33 | 0 |
| Arachnida | Araneae | Theridiidae | *Coleosoma* |  | 2075 | 127 |
| Arachnida | Araneae | Theridiidae | *Platnickina* | *Platnickina mneon* | 0 | 30 |
| Arachnida | Araneae | Thomisidae | *Xysticus* |  | 909 | 139 |
| Arachnida | Sarcoptiformes | Oppiidae |  |  | 2815 | 122 |
| Arachnida | Sarcoptiformes | Oribatellidae | *Oribatella* |  | 2916 | 28 |
| Arachnida | Sarcoptiformes | Phenopelopidae | *Peloptulus* |  | 856 | 0 |
| Arachnida | Sarcoptiformes | Scutoverticidae |  |  | 1203 | 0 |
| Arachnida | Trombidiformes | Bdellidae | *Cyta* |  | 18 | 0 |
| Arachnida | Trombidiformes | Eriophyidae | *Acaphylla* | *Acaphylla theavagrans* | 141 | 0 |
| Arachnida | Trombidiformes | Eupodidae |  |  | 125 | 0 |
| Arachnida | Trombidiformes | Tarsonemidae | *Polyphagotarsonemus* |  | 0 | 17 |
| Collembola | Entomobryomorpha | Entomobryidae |  |  | 45 | 194 |
| Collembola | Entomobryomorpha | Entomobryidae | *Entomobrya* |  | 65 | 11 |
| Collembola | Entomobryomorpha | Entomobryidae | *Homidia* | *Homidia sinensis* | 0 | 0 |
| Collembola | Entomobryomorpha | Tomoceridae | *Tomocerus* |  | 73 | 0 |
| Collembola | Neelipleona | Neelidae |  |  | 302 | 0 |
| Insecta |  |  |  |  | 15591 | 1064 |
| Insecta | Coleoptera | Cerambycidae | *Prionus* |  | 115 | 0 |
| Insecta | Coleoptera | Chrysomelidae |  |  | 557 | 0 |
| Insecta | Coleoptera | Chrysomelidae | *Cassida* |  | 7 | 0 |
| Insecta | Coleoptera | Coccinellidae |  |  | 0 | 0 |
| Insecta | Coleoptera | Coccinellidae | *Harmonia* | *Harmonia axyridis* | 58 | 0 |
| Insecta | Coleoptera | Dermestidae |  |  | 97 | 0 |
| Insecta | Coleoptera | Elmidae | *Riolus* |  | 4613 | 1348 |
| Insecta | Coleoptera | Latridiidae | *Cortinicara* | *Cortinicara gibbosa* | 116 | 0 |
| Insecta | Coleoptera | Oedemeridae |  |  | 2 | 186 |
| Insecta | Coleoptera | Scarabaeidae |  |  | 11478 | 0 |
| Insecta | Coleoptera | Scarabaeidae | *Anomala* |  | 3008 | 0 |
| Insecta | Coleoptera | Scydmaenidae | *Euconnus* |  | 2 | 0 |
| Insecta | Dermaptera | Forficulidae | *Forficula* |  | 50594 | 4600 |
| Insecta | Diptera |  |  |  | 3235 | 0 |
| Insecta | Diptera | Anthomyiidae | *Heterostylodes* |  | 2475 | 11 |
| Insecta | Diptera | Asilidae |  |  | 8661 | 0 |
| Insecta | Diptera | Asilidae | *Machimus* |  | 4357 | 0 |
| Insecta | Diptera | Asilidae | *Stenopogon* |  | 2079 | 1 |
| Insecta | Diptera | Cecidomyiidae |  |  | 2892 | 190 |
| Insecta | Diptera | Chironomidae |  |  | 148 | 0 |
| Insecta | Diptera | Chironomidae | *Tanytarsus* | *Tanytarsus formosanus* | 785 | 0 |
| Insecta | Diptera | Chironomidae | *Tanytarsus* | *Tanytarsus pollexus* | 1 | 0 |
| Insecta | Diptera | Culicidae |  |  | 3 | 0 |
| Insecta | Diptera | Culicidae | *Culex* | *Culex bahamensis* | 195 | 0 |
| Insecta | Diptera | Culicidae | *Culex* | *Culex quinquefasciatus* | 0 | 34 |
| Insecta | Diptera | Culicidae | *Culex* | *Culex tritaeniorhynchus* | 99 | 0 |
| Insecta | Diptera | Dolichopodidae | *Amblypsilopus* |  | 4139 | 0 |
| Insecta | Diptera | Drosophilidae | *Drosophila* |  | 1148 | 498 |
| Insecta | Diptera | Drosophilidae | *Leucophenga* |  | 4881 | 98 |
| Insecta | Diptera | Drosophilidae | *Scaptomyza* | *Scaptomyza pallida* | 9082 | 322 |
| Insecta | Diptera | Muscidae | *Muscina* | *Muscina angustifrons* | 620 | 0 |
| Insecta | Diptera | Muscidae | *Spilogona* |  | 1742 | 37 |
| Insecta | Diptera | Mycetophilidae |  |  | 48 | 0 |
| Insecta | Diptera | Phoridae | *Megaselia* |  | 12607 | 123 |
| Insecta | Diptera | Psychodidae |  |  | 0 | 0 |
| Insecta | Diptera | Psychodidae | *Psychoda* |  | 0 | 12 |
| Insecta | Diptera | Psychodidae | *Psychoda* | *Psychoda alternata* | 14 | 0 |
| Insecta | Diptera | Scatopsidae |  |  | 130 | 0 |
| Insecta | Diptera | Sciaridae |  |  | 552 | 38 |
| Insecta | Diptera | Sciaridae | *Bradysia* |  | 13 | 0 |
| Insecta | Diptera | Sciaridae | *Bradysia* | *Bradysia impatiens* | 44223 | 483 |
| Insecta | Diptera | Sciaridae | *Leptosciarella* |  | 1080 | 3 |
| Insecta | Diptera | Sphaeroceridae | *Pseudocollinella* |  | 26 | 0 |
| Insecta | Diptera | Tabanidae |  |  | 143 | 0 |
| Insecta | Diptera | Tachinidae | *Eumea* | *Eumea caesar* | 42 | 31 |
| Insecta | Diptera | Tachinidae | *Linnaemya* |  | 17 | 0 |
| Insecta | Hemiptera | Aleyrodidae | *Aleurocanthus* |  | 349 | 357 |
| Insecta | Hemiptera | Aphididae | *Amphorophora* |  | 38 | 0 |
| Insecta | Hemiptera | Cicadellidae | *Empoasca* |  | 81 | 0 |
| Insecta | Hemiptera | Cicadellidae | *Empoasca* | *Empoasca onukii* | 226 | 66 |
| Insecta | Hemiptera | Reduviidae | *Rhynocoris* | *Rhynocoris kumarii* | 0 | 127 |
| Insecta | Hymenoptera | Aphelinidae | *Encarsia* |  | 71 | 0 |
| Insecta | Hymenoptera | Braconidae |  |  | 11448 | 278 |
| Insecta | Hymenoptera | Eulophidae |  |  | 5 | 0 |
| Insecta | Hymenoptera | Eulophidae | *Baryscapus* |  | 519 | 27 |
| Insecta | Hymenoptera | Formicidae |  |  | 14715 | 418 |
| Insecta | Hymenoptera | Formicidae | *Nylanderia* |  | 121964 | 8455 |
| Insecta | Hymenoptera | Formicidae | *Tapinoma* |  | 495 | 0 |
| Insecta | Hymenoptera | Formicidae | *Lasius* | *Lasius platythorax* | 0 | 0 |
| Insecta | Lepidoptera | Autostichidae | *Autosticha* | *Autosticha modicella* | 142 | 0 |
| Insecta | Lepidoptera | Geometridae | *Ectropis* | *Ectropis grisescens* | 134 | 0 |
| Insecta | Lepidoptera | Geometridae | *Scopula* | *Scopula subpunctaria* | 1094 | 92 |
| Insecta | Lepidoptera | Gracillariidae | *Caloptilia* | *Caloptilia theivora* | 78 | 0 |
| Insecta | Orthoptera | Acrididae | *Acrida* | *Acrida cinerea* | 559 | 0 |
| Insecta | Orthoptera | Acrididae | *Gomphocerus* | *Gomphocerus sibiricus* | 10468 | 0 |
| Insecta | Thysanoptera | Thripidae | *Chaetanaphothrips* |  | 34 | 0 |
| Insecta | Thysanoptera | Thripidae | *Dendrothrips* | *Dendrothrips minowai* | 97 | 11 |

**Table S7.** The potential prey species of *O. alboannulata* in study regions.

| Class | Order | Family | Genus | Species | Individual number |
| --- | --- | --- | --- | --- | --- |
| Arachnida | Araneae | Agelenidae | *Agelena* | *unknown* | 1 |
| Arachnida | Araneae | Araneidae | *Araneus* | *Araneus ejusmodi* | 31 |
| Arachnida | Araneae | Araneidae | *Araneus* | *Araneus pentagrammicus* | 11 |
| Arachnida | Araneae | Araneidae | *Cyclosa* | *Cyclosa argenteoalba* | 16 |
| Arachnida | Araneae | Araneidae | *Eriovixia* | *Eriovixia cavaleriei* | 20 |
| Arachnida | Araneae | Araneidae | *Neoscona* | *Neoscona scylla* | 1 |
| Arachnida | Araneae | Araneidae | *Neoscona* | *Neoscona vigilans* | 10 |
| Arachnida | Araneae | Hahniidae | *Hahnia* | *Hahnia thorntoni* | 27 |
| Arachnida | Araneae | Linyphiidae | *Erigone* | *Erigone prominens* | 1 |
| Arachnida | Araneae | Linyphiidae | *Hylyphantes* | *Hylyphantes graminicola* | 2 |
| Arachnida | Araneae | Linyphiidae | *Ummeliata* | *Ummeliata insecticeps* | 1 |
| Arachnida | Araneae | Oxyopidae | *Oxyopes* | *unknown* | 14 |
| Arachnida | Araneae | Pisauridae | *Dolomedes* | *unknown* | 46 |
| Arachnida | Araneae | Salticidae | *Bristowia* | *Bristowia heterospinosa* | 138 |
| Arachnida | Araneae | Salticidae | *Evarcha* | *Evarcha albaria* | 25 |
| Arachnida | Araneae | Salticidae | *Myrmarachne* | *Myrmarachne gisti* | 17 |
| Arachnida | Araneae | Salticidae | *Orienticius* | *Orienticius vulpes* | 3 |
| Arachnida | Araneae | Salticidae | *Phintella* | *Phintella bifurcilinea* | 1 |
| Arachnida | Araneae | Salticidae | *Sibianor* | *unknown* | 43 |
| Arachnida | Araneae | Tetragnathidae | *Tetragnatha* | *Tetragnatha maxillosa* | 96 |
| Arachnida | Araneae | Theridiidae | *Chrosiothes* | *Chrosiothes sudabides* | 5 |
| Arachnida | Araneae | Theridiidae | *Coleosoma* | *Coleosoma blandum* | 301 |
| Arachnida | Araneae | Theridiidae | *Coleosoma* | *Coleosoma octomaculatum* | 26 |
| Arachnida | Araneae | Theridiidae | *Meotipa* | *Meotipa spiniventris* | 2 |
| Arachnida | Araneae | Theridiidae | *Meotipa* | *Meotipa vesiculosa* | 1 |
| Arachnida | Araneae | Theridiidae | *Paidiscura* | *Paidiscura subpallens* | 82 |
| Arachnida | Araneae | Theridiidae | *Phycosoma* | *Phycosoma sinica* | 21 |
| Arachnida | Araneae | Theridiidae | *Platnickina* | *Platnickina mneon* | 51 |
| Arachnida | Araneae | Theridiidae | *Theridion* | *Theridion submirabile* | 1 |
| Arachnida | Araneae | Thomisidae | *Ebrechtella* | *Ebrechtella tricuspidata* | 18 |
| Arachnida | Araneae | Thomisidae | *Oxytate* | *unknown* | 2 |
| Arachnida | Araneae | Thomisidae | *Xysticus* | *Xysticus croceus* | 433 |
| Arachnida | Araneae | Thomisidae | *Xysticus* | *Xysticus kurilensis* | 2 |
| Arachnida | Araneae | Trachelidae | *Orthobula* | *Orthobula crucifera* | 1 |
| Arachnida | Araneae | Trachelidae | *Trachelas* | *Trachelas sinensis* | 3 |
| Arachnida | Sarcoptiformes | Oppiidae | *unknown* | *unknown* | 68 |
| Arachnida | Trombidiformes | unknown | *unknown* | *unknown* | 7 |
| Chilopoda | Lithobiomorpha | Lithobiidae | *unknown* | *unknown* | 33 |
| Collembola | Entomobryomorpha | unknown | *unknown* | *unknown* | 124 |
| Collembola | Entomobryomorpha | Entomobryidae | *unknown* | *unknown* | 28 |
| Collembola | Neelipleona | Neelidae | *unknown* | *unknown* | 23 |
| Insecta | Coleoptera | unknown | *unknown* | *unknown* | 45 |
| Insecta | Coleoptera | Chrysomelidae | *unknown* | *unknown* | 2 |
| Insecta | Coleoptera | Coccinellidae | *unknown* | *unknown* | 2 |
| Insecta | Coleoptera | Coccinellidae | *Chiloconus* | *Chiloconus kuwanae* | 31 |
| Insecta | Coleoptera | Coccinellidae | *Serangium* | *Serangium japonicum* | 3 |
| Insecta | Coleoptera | Curculionidae | *unknown* | *unknown* | 2 |
| Insecta | Coleoptera | Staphylinidae | *Paederus* | *unknown* | 1 |
| Insecta | Corrodentia | unknown | *unknown* | *unknown* | 3 |
| Insecta | Dermaptera | Forficulidae | *Forficula* | *unknown* | 244 |
| Insecta | Diptera | unknown | *unknown* | *unknown* | 259 |
| Insecta | Diptera | Chloropidae | *Chlorops* | *Chlorops oryzae* | 10 |
| Insecta | Diptera | Chloropidae | *Elachiptera* | *unknown* | 38 |
| Insecta | Diptera | Drosophilidae | *Drosophila* | *Drosophila triauraria* | 33 |
| Insecta | Diptera | Drosophilidae | *Scaptomyza* | *Scaptomyza pallida* | 1469 |
| Insecta | Diptera | Tachinidae | *unknown* | *unknown* | 36 |
| Insecta | Hemiptera | unknown | *unknown* | *unknown* | 26 |
| Insecta | Hemiptera | Aleyrodidae | *Aleurocanthus* | *Aleurocanthus spiniferus* | 10 |
| Insecta | Hemiptera | Aphididae | *unknown* | *unknown* | 5 |
| Insecta | Hemiptera | Cicadellidae | *unknown* | *unknown* | 18 |
| Insecta | Hemiptera | Cicadellidae | *Empoasca* | *Empoasca onukii* | 324 |
| Insecta | Hemiptera | Pentatomidae | *Eysarcoris* | *Eysarcoris guttiger* | 54 |
| Insecta | Hymenoptera | Braconidae | *unknown* | *unknown* | 2 |
| Insecta | Hymenoptera | unknown | *unknown* | *unknown* | 13 |
| Insecta | Hymenoptera | Eulophidae | *unknown* | *unknown* | 2 |
| Insecta | Hymenoptera | Formicidae | *unknown* | *unknown* | 95 |
| Insecta | Hymenoptera | Ichneumonidae | *unknown* | *unknown* | 8 |
| Insecta | Lepidoptera | unknown | *unknown* | *unknown* | 32 |
| Insecta | Lepidoptera | Geometridae | *unknown* | *unknown* | 4 |
| Insecta | Lepidoptera | Geometridae | *Ectropis* | *Ectropis grisescens* | 57 |
| Insecta | Lepidoptera | Geometridae | *Scopula* | *Scopula subpunctaria* | 350 |
| Insecta | Lepidoptera | Lymantriidae | *unknown* | *unknown* | 1 |
| Insecta | Mantodea | unknown | *unknown* | *unknown* | 1 |
| Insecta | Neuroptera | unknown | *unknown* | *unknown* | 27 |
| Insecta | Odonata | unknown | *unknown* | *unknown* | 1 |
| Insecta | Orthoptera | Acrididae | *unknown* | *unknown* | 18 |
| Insecta | Orthoptera | Trigonidiidae | *Paratrigonidium* | *unknown* | 2 |
| Insecta | Thysanoptera | Thripidae | *unknown* | *unknown* | 2 |
| Insecta | Thysanoptera | Thripidae | *Dendrothrips* | *Dendrothrips minowai* | 56 |

**Supplementary Figures**


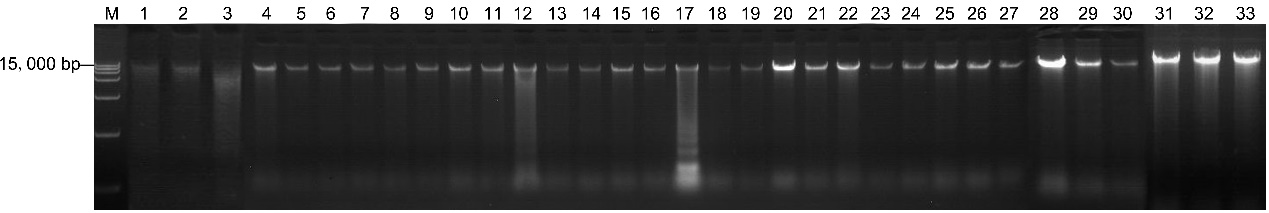


**Figure S1.** Agarose gel electrophoresis of the DNA extracted from *O. alboannulata*. Cropped gels are merged and displayed (the full length of each uncropped gel is 11.5 cm). M: DNA marker (DL15,000 DNA Marker-Takara). Lanes 1–30: individual DNA samples; Lanes 31–33: mixed DNA samples.
